# HDAC6 inhibition regulates substrate stiffness-mediated inflammation signaling in chondrocytes: HDAC6 regulates substrate stiffness-mediated inflammation signaling

**DOI:** 10.3724/abbs.2023144

**Published:** 2023-08-29

**Authors:** Yang Zhang, Godfred K Tawiah, Yanjun Zhang, Xiaohu Wang, Xiaochun Wei, Weiyi Chen, Xiaohong Qiao, Quanyou Zhang

**Affiliations:** 1 Department of Histology and Embryology Shanxi Medical University Jinzhong 030604 China; 2 College of Biomedical Engineering Taiyuan University of Technology Taiyuan 030024 China; 3 Department of Orthopaedics the Second Hospital of Shanxi Medical University Shanxi Key Laboratory of Bone and Soft Tissue Injury Repair Shanxi Medical University Taiyuan 030001 China; 4 Department of Orthopaedics Lvliang Hospital Affiliated to Shanxi Medical University Lvliang 033099 China

**Keywords:** chondrocyte, substrate stiffness, inflammation, HDAC6, primary cilia, matrix metabolism

## Abstract

Osteoarthritis (OA) is a chronic disease and is difficult to cure. Chondrocytes are highly mechanosensitive. Therefore, mechanical therapies have received attention as a therapeutic direction for OA. The stiffness, as a critical cue of the extracellular matrix (ECM), affects cell growth, development, and death. In this study, we use polydimethylsiloxane (PDMS) to create substrates with varying stiffness for chondrocyte growth, interleukin-1β (IL-1β) treatment to mimic the inflammatory environment, and Tubastatin A (Tub A) to inhibit histone deacetylase 6 (HDAC6). Our results show that stiff substrates can be anti-inflammatory and provide a better matrix environment than soft substrates. Inhibition of HDAC6 improves the inflammatory environment caused by IL-1β and coordinates with inflammation to spread the chondrocyte area and primary cilia elongation. Without IL-1β and Tub A treatments, the length of the primary cilia rather than frequency is stiffness-dependent, and their length on stiff substrates are greater than that on soft substrates. In conclusion, we demonstrate that stiff substrates, inflammation, and inhibition of HDAC6 enhance the mechanosensitivity of primary cilia and mediate substrate stiffness to suppress inflammation and protect the matrix.

## Introduction

The stiffness, as a key physical factor of the cell microenvironment, is well recognized to regulate different cell functions and cell-to-cell communication [ 1, 2]. It is well appreciated that stiffness has been shown to regulate chondrocyte morphology, phenotype and mechanical behavior [ 3, 4]. Chondrocytes are the only cell type in cartilage and are solely responsible for the production and maintenance of extracellular matrix (ECM). The local mechanical microenvironment of the matrix regulates the matrix homeostasis. The substrate stiffness-dependent mechanical deformation behavior and properties of chondrocytes may be essential for the function and survival of healthy chondrocytes *in vivo* [5]. The pericellular matrix (PCM) with defined mechanical properties surrounds each chondrocyte [6] and plays a critical role in regulating the mechanical microenvironment of chondrocytes [ 7, 8]. PCM stiffness significantly changes with the progress of osteoarthritis (OA). The stiffness of PCM is between 1 kPa and 205 kPa under different measurement techniques [ 5 , 9‒ 13]. In early osteoarthritic cartilage, PCM exhibits a stiffness 30% lower than that of normal cartilage [13], which leads to accelerated development of OA [14]. PCM stiffness can be reduced by 30%‒50% during the progression of OA [15], which regulates chondrocyte function-related spatial organization and biosynthetic activity [ 16 , 17].

In joint diseases such as OA, ECM becomes softer and loses its protective abilities, leading to changes in chondrocyte metabolism, inflammation, and cartilage degeneration [ 11 , 18, 19]. However, the precise mechanisms of how chondrocytes sense the environment and how this may result in OA are not well understood. Previous studies have indicated that abnormal activity, expression level, and distribution of histone deacetylases (HDACs) lead to the initiation and progression of OA [20]. As a member of the histone deacetylase family, HDAC6 does not bind to histones like other HDACs but selectively binds to α-tubulin and other substrates in the cytoplasm [ 21, 22]. A previous study reported that mechanical loading decreased inflammatory signaling by HDAC6 activation, which is associated with alterations in primary cilia in response to IL-1β [23], suggesting that HDAC6 is related to the inflammatory response. Tub A, a potent and highly selective HDAC6 inhibitor, has been reported to provide a therapeutic effect for several diseases [ 22, 24, 25]. Inhibition of HDAC6 with Tub A has been reported to reduce cartilage damage in experimental OA [ 26, 27]. Other results showed that treatment with Tub A significantly improves OA and inhibits the level of HDAC6 in chondrocytes, thereby activating autophagy and cell survival and reducing ECM degradation [26]. Therefore, HDAC6 inhibition by Tub A may be a feasible strategy for the management of OA. Although it has been reported that inhibiting HDAC6 can treat OA, the relationship among substrate stiffness, inflammatory signals and HDAC6 remains unknown.

The tubulin deacetylase activity of HDAC6 *in vivo* and *in vitro* was first reported by Hubbert *et al*. [21]. Furthermore, a previous study confirmed that the overexpression of HDAC6 promotes microtubule-dependent cell movement, suggesting that HDAC6 plays a role in regulating actin networks and microtubule dynamics and stability [28]. The mechanobiological behavior of chondrocytes is influenced by the matrix mechanical microenvironment [29]. Chondrocytes have diverse types of mechanosensors, such as mechanosensitive ion channels, integrins, and primary cilia. However, how chondrocytes sense and response to the mechanical microenvironment with mechanosensitive apparatus remains elusive. The primary cilium is now believed to be a multifunctional antenna that detects alterations in the extracellular microenvironment [ 30‒ 32]. The primary cilium consists of a membrane-coated axoneme that protrudes from the cell surface into the extracellular microenvironment and an intracellular basal body [ 33‒ 35]. Disruptions to primary cilia function have been shown to result in abnormalities in weight-bearing cartilage [ 36‒ 38]. HDAC6 is enriched within cilia and modulates cilia resorption through deacetylation and polymerization of ciliary tubulin [ 39‒ 41]. However, how HDAC6 mediates matrix stiffness in response to inflammation and chondrocyte primary cilia has not been reported.

In the present study, we hypothesized that: (i) substrate stiffness affects the response of chondrocytes to inflammation; (ii) HDAC6 influences the inflammatory response of chondrocytes on different substrate stiffness; and (iii) the spreading and length of primary cilia of chondrocytes are regulated by inflammation, HDAC6 and substrate stiffness. To test these hypotheses, we engineered PDMS substrates with different stiffness to simulate different matrix mechanical microenvironments, created an inflammatory environment with IL-1β and inhibited HDAC6 with Tub A.

## Materials and Methods

### PDMS matrix preparation

Curing agents were mixed with PDMS prepolymer (Dow Corning, Beijing, China) in mass ratios of 1:10 (stiff), 1:50 (medium) and 1:70 (soft). Once mixed, the PDMS was centrifuged in a bench-top centrifuge to remove air bubbles and then either poured into 35-mm petri dishes or confocal dishes to create 1-mm thick films. All PDMS matrices were cured at 70°C for 6 h. Before seeding cells, PDMS matrices were oxidized in an oxygen plasma cleaner (SBC-12; KYKY Technology Co., Ltd, Beijing, China), functionalized with rat type I collagen (0.036 mg/mL; Shengyou Biotechnology, Hangzhou, China) at 4°C overnight and sterilized by UV irradiation for 45 min [42].

### Isolation of primary chondrocytes and treatment

Five- to six-day-old mice (C57Bl/6) were used in this study. All procedures were approved by the Animal Ethics Committee of Taiyuan University of Technology, and the animal experiments were conducted under the International Guidelines and Standards on Animal Welfare. The mice were sacrificed under general anesthesia, and the femoral condyles and tibial plateau were isolated from the hind limbs. The pieces of cartilage were incubated in the collagenase D digestion solution (3 mg/mL) for 45 min at 37°C in an incubator with 5% CO _2_. After 45 min, the new collagenase D digestion solution was replaced and incubated for another 45 min in the same environment. Then, the cartilage pieces were placed in a new Petri dish with collagenase D solution (0.5 mg/mL) and incubated overnight at 37°C as previously described [43]. Then, cells were isolated by filtering through a 40-μm cell strainer and washed twice with fresh Dulbecco’s modified Eagle’s medium (DMEM; BOSTER, Wuhan, China). Cells were resuspended in DMEM supplemented with 10% fetal calf serum and 1% penicillin/streptomycin and were grown on substrates of different stiffness at 37°C with 5% CO _2_. Meanwhile, Tub A (HY-13271A; MedChemExpress, Monmouth Junction, USA) was used at different concentrations (10 nM, 100 nM, 500 nM, 1 μM, 5 μM, 10 μM, and 25 μM) to inhibit HDAC6.

### Measurement of nitrite (NO) and PGE2 release and HDAC6 activity

NO release was detected using the Griess assay kit (S0021S; Beyotime Biotechnology, Haimen, China). An immunoassay kit (KGE004B, R&D Systems, Minneapolis, USA) was used to quantify PGE2 release. HDAC6 activity was measured using a commercial HDAC6 fluorometric assay kit (ab284549; Abcam, Cambridge, UK).

### Immunofluorescence microscopy

Chondrocytes were fixed with 4% paraformaldehyde for 20 min, permeabilized with 0.5% Triton X-100/PBS for 5 min and blocked with 1% BSA for 1 h. Samples were incubated with polyclonal rabbit anti-Arl13b antibody (1:500; 17711-1-AP; Proteintech, Wuhan, China) overnight at 4°C. Then, the cells were washed five times with PBST before incubation with goat anti-rabbit Alexa 647 (1:2000; Invitrogen, Carlsbad, USA) for 1 h at room temperature. After 1 h of light-protected incubation, the samples were washed five times. Then, the samples were incubated with Alexa Fluor 488 phalloidin (Thermo Scientific, Waltham, USA) for 20 min at room temperature in the dark. Samples were washed five times and counterstained with DAPI. Finally, cells were photographed with a confocal microscope (Leica, Wetzlar, Germany).

### Western blot analysis

Total protein extraction was carried out by lysing cells with 1× SDS loading buffer and denatured at 95°C for 5 min. The sample volume per lane was 10 μL. The protein samples were separated by SDS-PAGE and then transferred onto PVDF membranes (Millipore, Billerica, USA). After being blocked and washed, the membranes were incubated overnight at 4°C with primary antibodies, followed by incubation with the corresponding secondary antibodies. Finally, the membrane was exposed with ECL developer (CW0049M; CWbio, Beijing, China) and processed with ImageJ software ( https://imagej.nih.gov/ij/). The primary antibodies used were as follows: anti-GAPDH (1:1000; CWbio), anti-HDAC6 (1:1000; 12834-1AP; Proteintech), anti-acetylated tubulin (1:1000; T7451; Sigma, St Louis, USA), anti-α-tubulin (1:1000; T6199; Sigma), anti-collagen II (1:1000; ab34712; Abcam), anti-MMP13 (1:1000; Wanleibio, Shenyang, China), and anti-SOX9 (1:1000; ab182579; Abcam). The secondary antibodies were goat anti-mouse IgG (1:4000; CWbio) and goat anti-rabbit IgG (1:4000; CWbio).

### Statistical analysis

Data are presented as the mean±standard deviation. Statistical differences between the groups were estimated using one-way analysis of variance (ANOVA) followed by Tukey’s post hoc test. When two variables were involved, two-way ANOVA was used, and a significant interaction was interpreted using Tukey’s post hoc multiple comparisons test. The analysis software was GraphPad Prism 9. *P* values are presented as follows: * *P*<0.05, ** *P*<0.01, *** *P*<0.001, **** *P*<0.0001, and ‘ns’ means no significant difference.

## Results

### Chondrocytes in response to IL-1β on varying substrate stiffness

**Figure 1:**
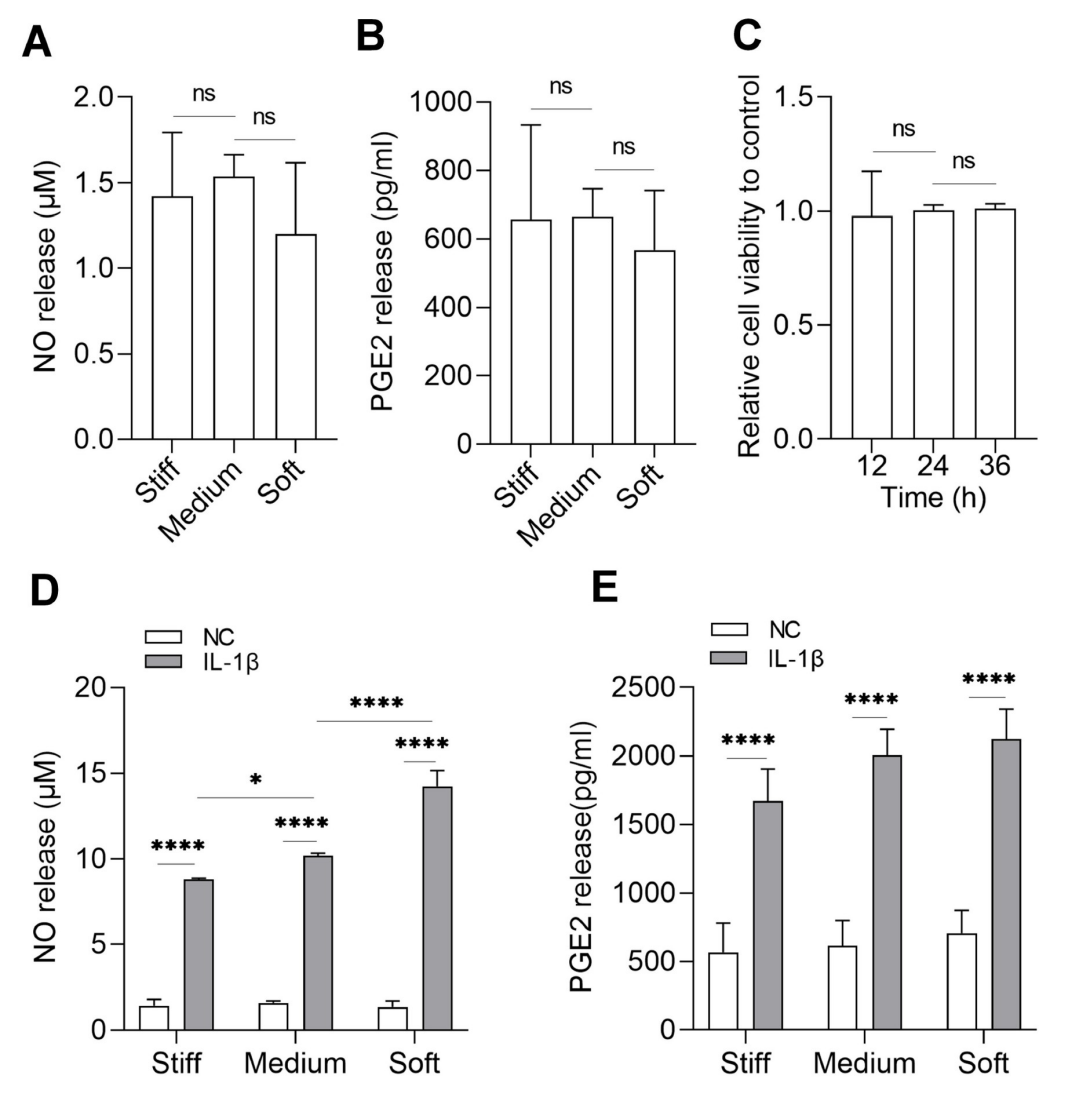
Figure 1 Chondrocytes response to IL-1β on varying substrate stiffness (A) NO and (B) PGE2 release without IL-1β treatment were measured on stiff, medium, soft substrates. (C). Chondrocytes were cultured in culture dishes and treated with 10 ng/mL IL-1β for 12 h, 24 h and 36 h. Cell activity was measured by CCK 8 assay. (D) NO and (E) PGE2 release with IL-1β (10 ng/mL) treatment for 12 h, and measured on stiff, medium, and soft substrates. All samples labelled with IL-1β were treated with IL-1β for 12 h. The sample of NC was without any treatment. n=3. ns, not significant; *P<0.05, and ****P<0.0001.

We treated chondrocytes with IL-1β to mimic the inflammatory environment and examined the effect of different substrate stiffness on the response to IL-1β treatment in chondrocytes. First, control group (NC) without IL-1β treatment for 12 h was set up, and the results showed that NO and PGE2 release did not differ on substrates with different stiffness ( Figure 1A,B). Incubation of chondrocytes with IL-1β (10 ng/mL) had no effect on cell activity within 36 h ( Figure 1C). IL-1β (10 ng/mL) treatment of chondrocytes for 12 h was selected. When chondrocytes were cultured on PDMS of three substrates with IL-1β for 12 h, NO and PGE2 release were significantly increased in each PDMS, which further confirmed that it is feasible to induce inflammation with IL-1β treatment. However, compared with that on the soft substrates, the increase in NO release on the stiff substrates was lower ( Figure 1D). Another inflammatory mediator, PGE2 release, was also increased notably in each substrate stiffness; however, the difference in PGE2 release on stiff, medium, and soft substrates was not greater than the difference in NO release ( Figure 1 E).

### The level of matrix metabolism in response to inflammation on different substrate stiffness

**Figure 2:**
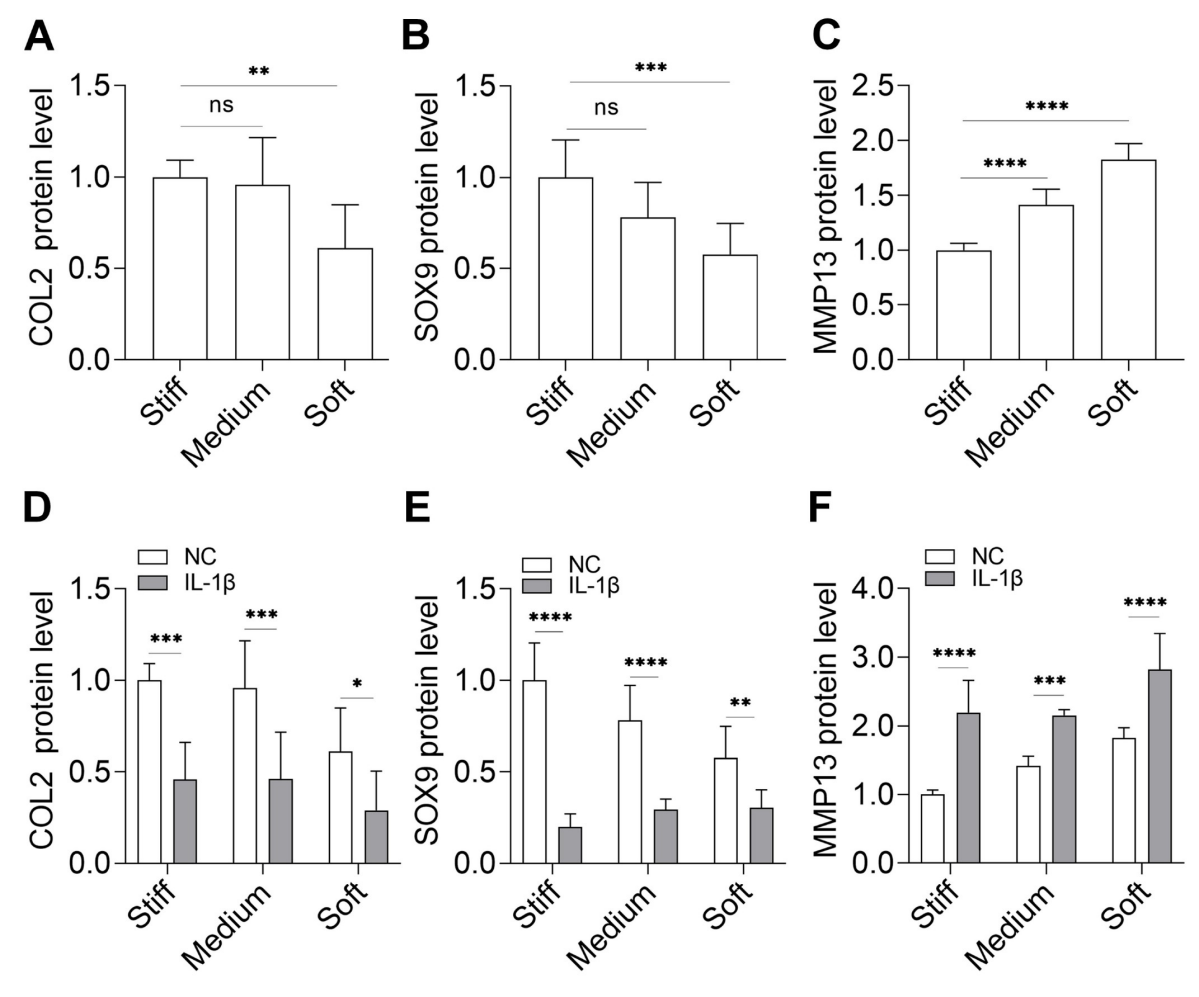
Figure 2 The level of ECM metabolism in response to inflammation on different substrate stiffness (A‒C) Western blot analysis of COL2, SOX9 and MMP13 expressions in samples without IL-1β treatment. (D‒F) Western blot analysis of COL2, SOX9 and MMP13 expressions in samples with or without IL-1β treatment. n=3. ns, not significant; * P<0.05, **P<0.01, ***P<0.001, and ****P<0.0001.

COL2 and SOX9 reflect a certain level of matrix synthesis, and MMP13 is one of the indicators of matrix degradation. To determine whether substrate stiffness affects ECM metabolism, we analyzed the protein expression levels of COL2, SOX9 and MMP13 in samples from different substrate stiffness by western blot analysis. The expression levels of COL2 ( Figure 2A) and SOX9 ( Figure 2B) on soft substrates were lower than those on stiff substrates, while MMP 13 expression was higher on soft substrates than on stiff substrates ( Figure 2C).

To detect the response to inflammation, we also analyzed these protein expressions, and the results showed that IL-1β treatment for 12 h decreased the expression levels of COL2 and SOX9, ultimately at the same level. However, the decrease was greater on the stiff substrates than on soft substrates ( Figure 2D,E). At the same time, the expression level of MMP13 was higher than that in samples without IL-1β treatment. Additionally, the increase was greater on the stiff substrates than on soft substrates ( Figure 2F).

### HDAC6 inhibition regulates the substrate stiffness-mediated IL-1β response and ECM metabolism

**Figure 3:**
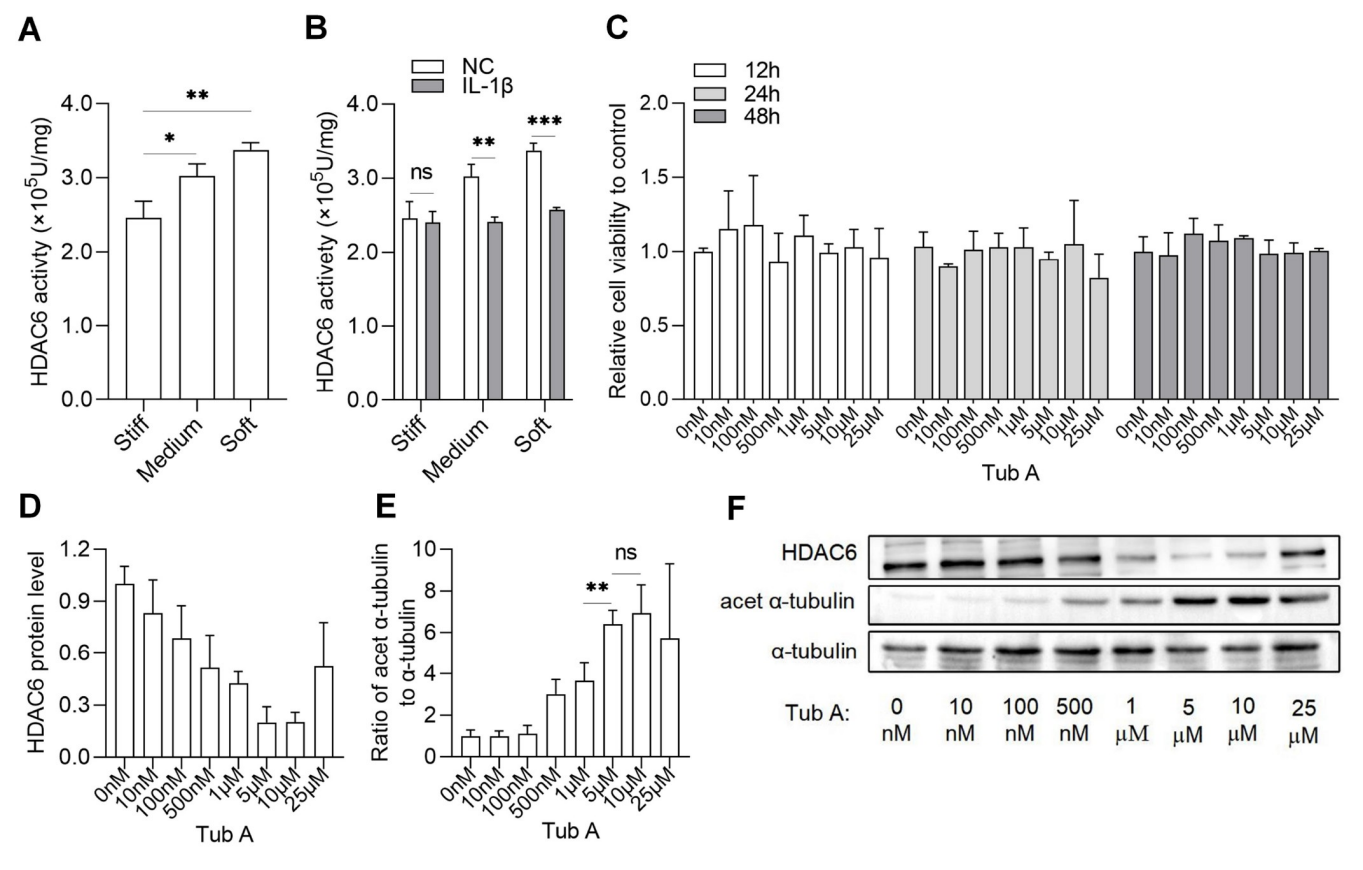
Figure 3 The effect of substrate stiffness and IL-1β on HDAC6 activity (A,B) Chondrocytes were cultured on stiff, medium, and soft substrates with or without IL-1β treatment for 24 h and HDAC6 activity was measured using the corresponding assay kit. (C) Chondrocytes were treated with various concentrations of Tub A (0 nM, 10 nM, 100 nM, 500 nM, 1 μM, 5 μM, 10 μM, 25 μM) for 12 h, 24 h, 36 h, and the cell activity was measured by CCK 8 assay in 96-well plates. (D) Western blot analysis of HDAC6 in chondrocytes treated with Tub A at various concentrations. (E) Western blot analysis of Acet α-tubulin and α-tubulin in chondrocytes treated with Tub A at various concentrations, and the ratio of acetylated-α-tubulin to total α-tubulin was calculated. (F) The western blot bands of (D‒E). n=3. ns, not significant; *P<0.05, **P<0.01, and ***P<0.001.

To investigate the role of HDAC6 in response to IL-1β and ECM metabolism on different substrate stiffness, we first measured HDAC6 activity. Our results showed that without IL-1β treatment, HDAC6 activity on soft substrates was higher than that on stiff and medium substrates ( Figure 3A). Interestingly, we observed low HDAC6 activity, high levels of COL2 and SOX9 protein and low level of MMP13 on stiff substrates and high HDAC6 activity, low levels of COL2 and SOX9 protein and high level of MMP13 on soft substrates ( Figures 2A‒C and 3A). In an inflammatory environment, HDAC6 activity was decreased on soft substrate stiffness ( Figure 3B). However, the relationship described above was disturbed, and the decrease in HDAC6 activity did not lead to higher levels of COL2 and SOX9 or lower level of MMP13 ( Figure 2E‒G). On stiff substrates, HDAC6 activity did not notably decrease, but the levels of COL2 and SOX9 were decreased, and the release of NO and PGE2 was eventually increased. These results suggested that the mode of action of HDAC6 may be inconsistent between inflammatory and noninflammatory environments, and for stiff substrates, the response to inflammation may not be directly dependent on HDAC6 activity.

We further explored the relationship between HDAC6 and matrix metabolism and the release of NO and PGE2 in an inflammatory environment. We used Tub A to inhibit HDAC6. When the concentrations of Tub A were 0 nM, 10 nM, 100 nM, 500 nM, 1 μM, 5 μM, 10 μM, and 25 μM and the chondrocytes were treated for 12 h, 24 h and 36 h, the cell activity was not significantly different ( Figure 3C). We selected 24 h as the Tub A treatment time. Meanwhile, the expression level of HDAC6 ( Figure 3D,F) and acetylation rates of α-tubulin, a substrate for HDAC6 ( Figure 3E,F) were measured by western blot analysis to find the optimal inhibitory concentration. The results showed that 10 μM Tub A was already able to achieve better inhibition of HDAC6. We selected 10 μM as the Tub A treatment concentration.

**Figure 4:**
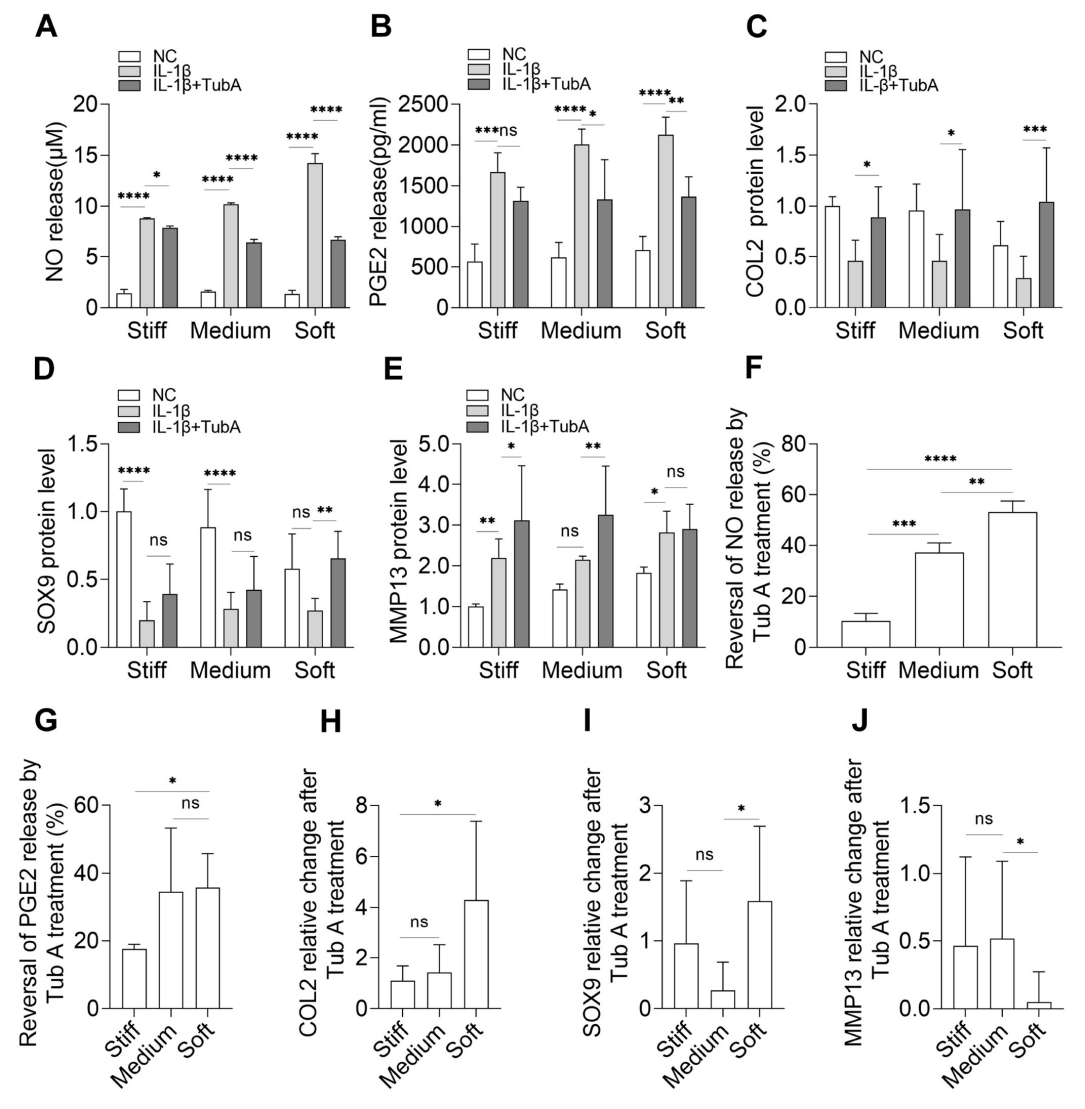
Figure 4 HDAC6 inhibition regulates substrate stiffness-mediated IL-1β response and ECM metabolism (A) NO and (B) PGE2 release with the treatment of control (without IL-1β and Tub A), IL-1β and IL-1β+Tub A on different substrates, and all samples labelled with ‘IL-1β+Tub A’ were first treated with Tub A for 24 h, followed by treatment with IL-1β for 12 h. The protein level of (C) COL2, (D) SOX9 and (E) MMP13 with the treatment of NC, IL-1β and IL-1β+Tub A on varying substrates. (F) Reversal of NO release by Tub A treatment=[(IL-1β+Tub A‒IL-1β)/IL-1β] ×100%, and data came from (A). (G) Reversal of PGE2 release by Tub A treatment=[(IL-1β+Tub A‒IL-1β)/IL-1β] ×100%, and data came from (B). (H) Relative COL2 change after Tub A treatment=(IL-1β+Tub A‒IL-1β) / IL-1β, and data came from (C). (I) Relative SOX9 change after Tub A treatment=(IL-1β+Tub A‒IL-1β)/IL-1β, and data came from (D). Relative MMP13 change after Tub A treatment=(IL-1β+Tub A‒IL-1β)/IL-1β, and data came from (E). n=3. ns, not significant; *P<0.05, **P<0.01, *** P<0.001, and ****P<0.0001.

Chondrocytes were treated with Tub A for 24 h and then with IL-1β for 12 h. The results showed that the levels of NO ( Figure 4A) and PGE2 ( Figure 4B) release were reversed, although the difference at the final level was not apparent. The reversal amplitudes were different on varying substrates, and the degree on soft substrates was larger than that on stiff substrates ( Figure 4F,G). These results were consistent with the changes in HDAC6 activity on stiff substrates ( Figure 3B). Perhaps the response of NO and PGE2 release on stiff substrates maybe independent of HDAC6. COL2 expression was also reversed ( Figure 4C) and more obvious on soft substrates ( Figure 4H) and SOX9 ( Figure 4D,I). For MMP13 expression, with the inhibition of HDAC6, MMP13 level was higher on stiff and medium substrates than on soft substrates ( Figure 4E,J).

### HDAC6 cooperates with IL-1β to regulate cell size on substrates with varying stiffness

**Figure 5:**
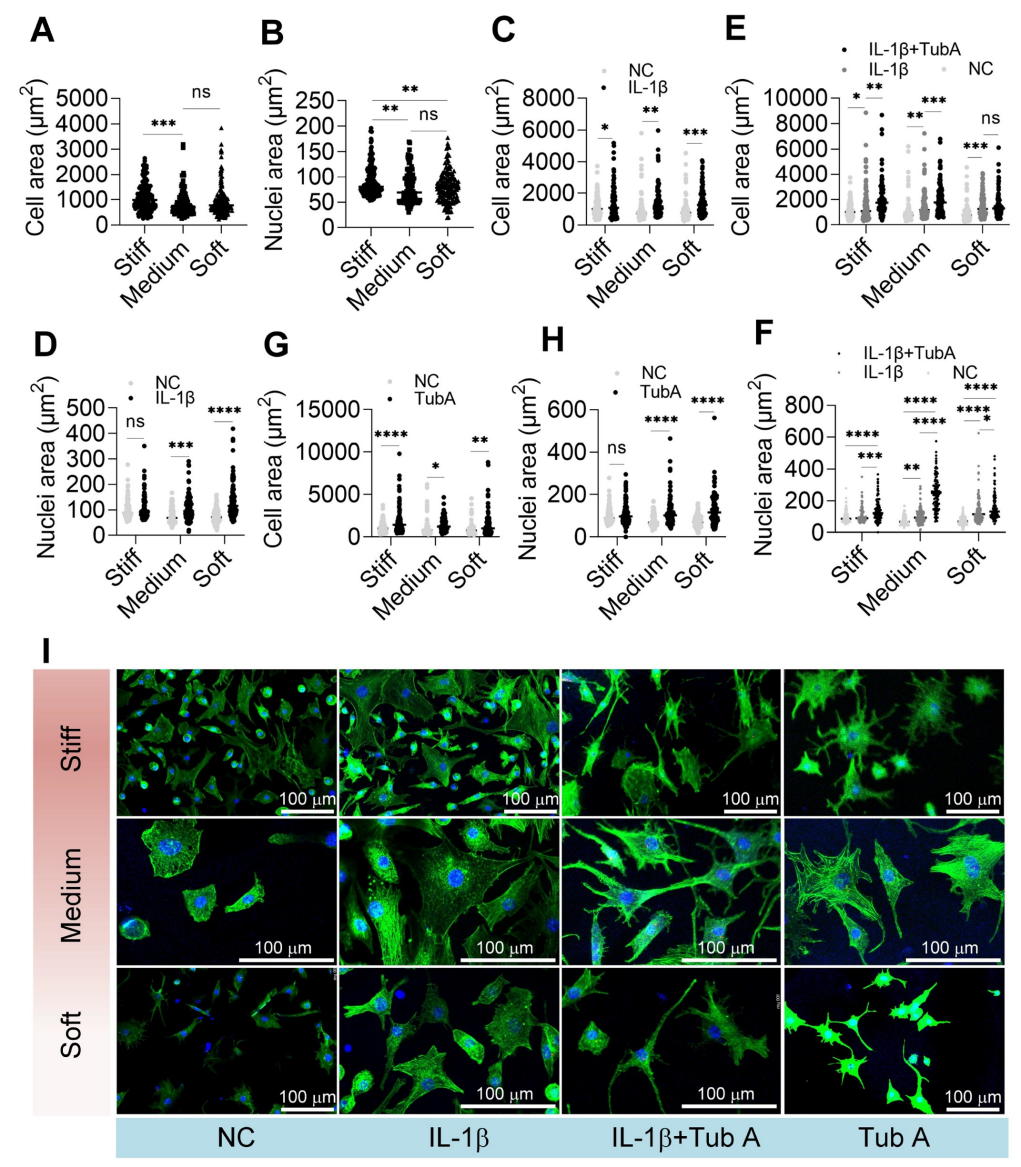
Figure 5 HDAC6 cooperates with IL-1β to regulate cell size on substrate with varying stiffness (A) Cell area and (B) nucleus area on substrates with varying stiffness without any treatment. (C) Cell area and (D) nucleus area on substrates with varying stiffness with IL-1β treatment compared with (A) and (B). (E) Cell area and (F) nucleus area on substrates with varying stiffness with IL-1β+Tub A treatment compared with (C) and (D). (G) Cell area and (H) nucleus area on substrates with varying stiffness with TubA treatment compared with (A) and (B). (I) Morphology of the cells under the different treatments. Blue: DAPI staining; green: phalloidin staining. n>100. ns, not significant; *P<0.05, **P<0.01, ***P<0.001, and ****P<0.0001.

To investigate the relationship between substrate stiffness and the spread area of chondrocytes and nuclei, we measured the spread areas of chondrocytes and nuclei with ImageJ software. The results showed that compared with those on soft substrates, chondrocytes on stiff substrates were relatively larger in both nuclear and spread areas ( Figure 5A,B). In an inflammatory environment, the spread areas of chondrocytes and nuclei were larger on stiff, medium, and soft substrates, especially on soft substrates ( Figure 5C,D). According to the above results, Tub A treatment reduced the inflammatory response of chondrocytes ( Figure 4). We further investigated the effect of HDAC6 on cell spreading, and the results showed that inhibiting HDAC6 did not reduce the larger cell area brought about by IL-1β treatment but instead increased the cell area ( Figure 5E,F). In addition, we treated chondrocytes only with Tub A, and the chondrocyte area also became larger ( Figure 5G,H). These results indicated that HDAC6 cooperates with IL-1β to regulate cell area on different substrate stiffness.

Although both IL-1β and Tub A treatment enlarged chondrocytes, they were slightly different in cell morphology, with Tub A treatment showing more excessive dendrites compared to the spread of IL-1 β-treated cells ( Figure 5I).

### Primary cilia respond to IL-1β and Tub A treatments on different substrate stiffness

**Figure 6:**
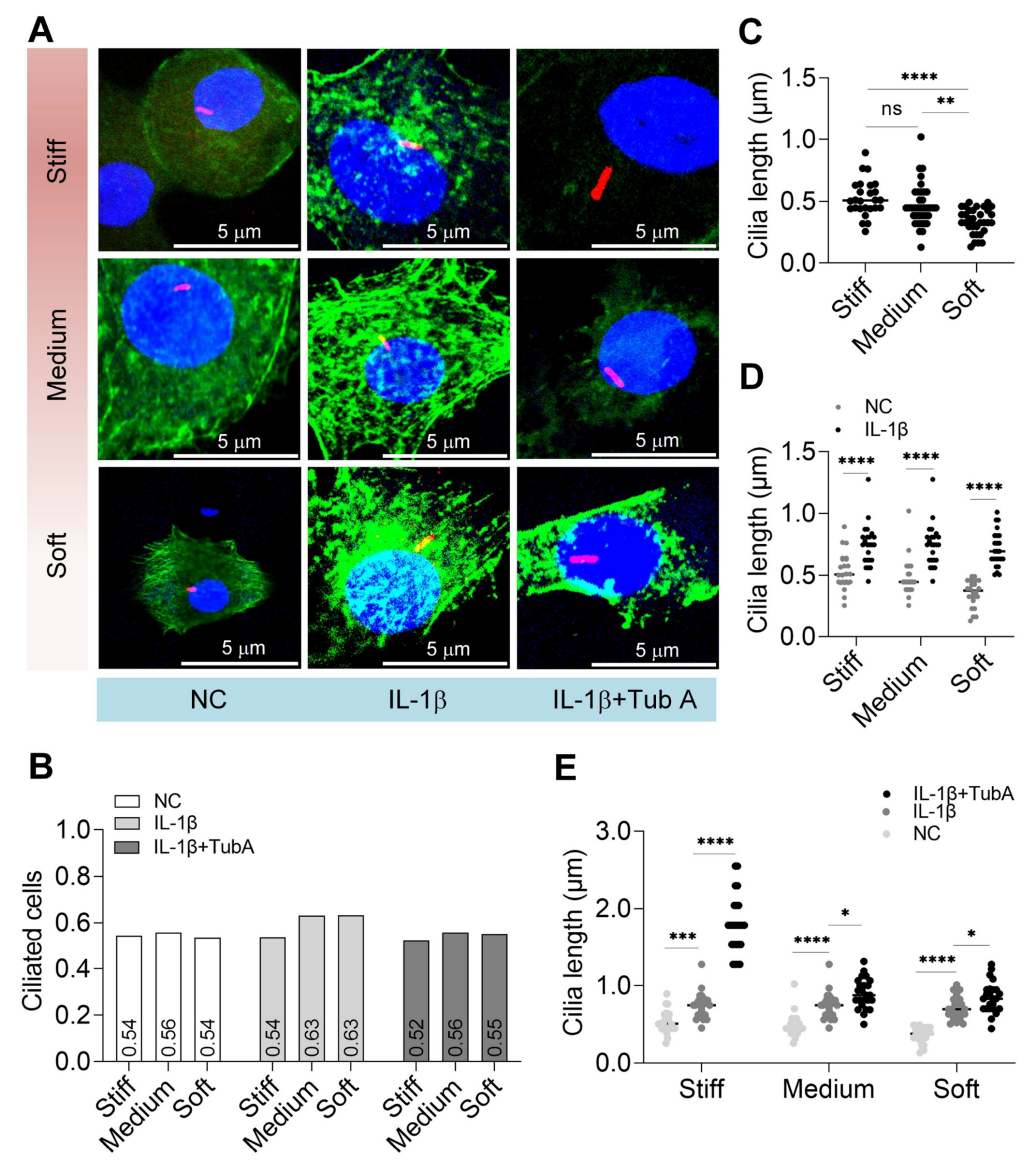
Figure 6 Primary cilia respond to IL-1β and Tub A on different substrate stiffness (A) Immunofluorescent staining of the chondrocytes on PDMS of stiff, medium, and soft substrates. Blue: DAPI staining; green: phalloidin staining; red: primary cilia. (B) The frequency of primary cilia occurred (cells with primary cilia/total number of cells, n>40). (C) The length of primary cilia on stiff, medium, and soft substrates (n=24). (D) The length of primary cilia was compared between the control and IL-1β treatment on stiff, medium, and soft substrates (n=24). (E) Comparison of the length of primary cilia among control, IL-1β treatment only, and both TubA and IL-1β treatment, on stiff, medium and soft substrates (n=24). ns, not significant; *P<0.05, **P<0.01, ***P<0.001, and ****P<0.0001

Primary cilium is as a functional antenna for sensing the microenvironment. We also focused on the response of primary cilia to IL-1β and Tub A treatments on different substrate stiffness ( Figure 6A). Within the chondrocytes in our statistics, the prevalence of primary cilia ranged between 0.5227 and 0.6327 ( Figure 6B). The length of primary cilia on soft substrates was shorter than that on stiff and medium substrates ( Figure 6C). When we treated chondrocytes with IL-1β, the primary cilia were all significantly longer; however, the range of primary cilia elongation on soft substrates was greater than that on stiff and medium substrates ( Figure 6D). To explore the effect of HDAC6 on inflammatory stimuli, we first inhibited HDAC6 with Tub A and then treated chondrocytes with IL-1β. The results showed that inhibition of HDAC6 amplified the effect of IL-1β treatment on cilia elongation ( Figure 6E).

## Discussion

In this study, we examined the inflammatory response of different substrate stiffness to IL-1β and the participation of HDAC6 and primary cilia. The ECM provides the mechanical microenvironment, so substrate stiffness is critical to the state of chondrocytes [44]. It has been reported that the ECM stiffness of OA differs from that of healthy cartilage [15]. In this study, we first focused on the relationship between substrate stiffness and inflammatory stimuli. The levels of NO [45] and PGE2 [46], as markers of the proinflammatory mediators, are higher in osteoarthritic joints, and it is well known that in OA, ECM metabolism is unbalanced, with levels of matrix degradation greater than synthesis. We added IL-1β to induce inflammation to simulate the inflammatory environment, and the results verified that the inflammatory environment decreased the level of matrix synthesis and increased the levels of matrix degradation ( Figure 2) and the release of NO and PGE2 ( Figure 1). However, the range of decrease in varying substrate stiffness was discrepant, and we found that at least in the short term (12 h), the level of NO release on stiff substrates was lower than that on soft substrates, which indicated that stiff substrates are conducive to reducing inflammation. Meanwhile, in an inflammatory environment, the level of matrix synthesis was slightly higher and matrix degradation was slightly lower on stiff substrates ( Figures 1 and 2). We also focused on chondrocytes on substrate stiffness without IL-1β treatment. There was no difference in inflammatory signals among different substrate stiffness within 12 h, indicating that the untreated chondrocytes were indeed in a noninflammatory state ( Figure 1A,B). Under noninflammatory environment, the matrix metabolism was different on substrates with varying stiffness, and on stiff substrates, the synthesis level was higher and the degradation level was lower ( Figure 2B‒D). Thus, chondrocytes on soft substrates may be more unstable, and soft substrates are more similar to the ECM of OA [15]. Together, these results suggest that stiff substrates are advantageous for chondrocyte survival.

The spreading area and morphology of chondrocytes objectively reflect the response of cells to the environment [47]. This study showed that stiff substrates, IL-1β stimulation, and tubulin acetylation can help spread chondrocytes. Substrate stiffness is a key regulator of cell area spreading, with reduced traction force generation of cells, smaller cell area, and more circular cells on soft substrates and vice versa on stiff substrates [48]. Interestingly, although stiff substrates are more conducive to chondrocyte spreading than soft substrates, the change in the spreading area of chondrocytes on soft substrates is more significant with IL-1β treatment ( Figure 5).

Primary cilia mediate the transduction of mechanical stress and are involved in many physiological and developmental processes [ 49‒ 51]. The acetylation of α-tubulin is essential for the assembly of primary cilia [52]. Longer primary cilia enhance mechanotransduction of chondrocytes [53]. Our results also verified that the length of primary cilia on stiff substrates was larger than that on soft substrates ( Figure 6C). Inflammation stimuli enhance the length of primary cilia [23], and we demonstrated that similar to cell spreading, inflammation was able to more significantly increase the ciliary length on soft substrates. We observed that the length of primary cilia and the spreading of chondrocytes showed the same changing pattern and a large spreading area when the ciliary became longer ( Figures 5 and 6). One previous study reported that controlling the spreading of cells regulates basal position and primary cilia growth [54]. However, the effect of IFT88 (a key protein of primary cilia) gene silencing on cellular spreading is negligible [55]. In summary, on our future work we will explore the relationship between ciliary microtubules and the cellular cytoskeleton, and determine whether cytoskeleton disruption affects primary cilia (or whether primary cilia disruption impacts the cellular cytoskeleton) and the underlying mechanisms.

Previous reports have also suggested the anti-inflammatory, anti-rheumatic [56], and anti-hepatitis C activities [57] of Tub A. However, its anti-inflammatory effect on varying substrate stiffness in chondrocytes has not yet been studied. Our results showed that the inhibition of HDAC6 can indeed play roles in anti-inflammation and ECM protection in chondrocytes ( Figure 4). In addition, combined with the HDAC6 activity test, HDAC6 did not appear to play equal roles in both inflammatory and noninflammatory environments. The response of stiff substrates to inflammation does not appear to be directly dependent on HDAC6 activity. Regarding this point, more evidence is needed. Moreover, our results showed that HDAC6 activity changed significantly on the soft substrates when treated with IL-1β ( Figure 3B), and in combination with the above results, it changed more on the soft substrates than on the stiff substrates ( Figures 4, 5C,D, and 6D). We speculated that the effects of soft substrates on HDAC6 activity are greater than those of stiff substrates; that is, HDAC6-regulated functions of chondrocytes on soft substrates were more active.

HDACs are a family of 18 enzymes that function to deacetylate histone proteins modulating chromosome structure, thus contributing to the regulation of gene transcription. HDAC6 modulates the acetylation and deacetylation balance of microtubules, and overexpression of HDAC6 promotes microtubule-dependent cell motility [ 21 , 40]. Inflammatory stimuli increase the length of primary cilia, and inhibition of HDAC6 partially protects against inflammation. We investigated the relationship between HDAC6 and primary cilia and cell spreading, and unexpectedly, although inhibition of HDAC6 reduced the inflammatory status of chondrocytes, it synergized with the enlargement of chondrocytes brought about by inflammatory stimuli, especially on stiff substrates. However, as the chondrocyte spread area increased, the morphology of chondrocytes also changed, and chondrocytes treated with HDAC6 inhibition developed more antennae ( Figure 6). Perhaps after inhibition of HDAC6, the cytoskeleton presents a state of acetylation, and microtubules tends to assemble and extend more in a way that inevitably leads to an imbalance in the stress state of the original cytoskeleton and thus more antennae. According to our results, inhibition of HDAC6 promotes the assembly and extension of microtubules and protects against inflammation. However, we did not determine whether HDAC6 mediates the assembly of microtubules against inflammation, which is the limitation of this study, and we need to go deep into the relationship between HDAC6 and both microtubules and inflammation combined with the domains of HDAC6.

In conclusion, as a microenvironment, substrate stiffness plays an important role in regulating the function of chondrocytes, and we showed that stiff substrates provide a better environment than soft substrates and reduce the inflammatory response. In the absence of perturbation of chemicals or other physical factors, substrate stiffness affects cell spreading and ciliary length in a stiffness-specific manner. When Tub A was used to inhibit HDAC6, a deacetylate histone protein that modulates microtubules, the inflammatory response was reduced and coordinated with inflammatory stimulation to regulate the cell spreading area and ciliary length. We tended to think that the inflammatory environment forces passive elongation of primary cilia to improve their mechanotransduction effects and then allows chondrocytes to reach the optimal state to adapt to inflammation; however, when HDAC6 is inhibited, primary cilia improve mechanotransduction by active elongation to adjust their most appropriate state. Thus, we believe that the assembly and depolymerization of primary cilia does not necessarily completely represent the good or bad condition of matrix metabolism. In addition to changes in chondrocyte area, we observed that chondrocytes treated with Tub A presented more dendritic antennae. Both IL-1β treatment and Tub A treatment can spread cells. From a microtubule composition perspective, this also supports the possibility that primary cilia elongate in a possible active and passive manner. The mechanism by which HDAC6 is involved in regulating the primary and cellular cytoskeleton of chondrocytes on substrates with varying stiffnesses will be the focus of our future studies. The current study provides a basis for finding novel strategies for mechanotherapy.
